# Exploring the Photophysical and Mechanical Behavior of Fluorescent Metal–Organic Framework Monoliths

**DOI:** 10.1021/acs.chemmater.4c00963

**Published:** 2024-08-20

**Authors:** Michele Tricarico, Samraj Mollick, Vishal Kachwal, Dylan A. Sherman, Jin-Chong Tan

**Affiliations:** Multifunctional Materials and Composites (MMC) Laboratory, Department of Engineering Science, University of Oxford, Parks Road, Oxford OX1 3PJ, U.K.

## Abstract

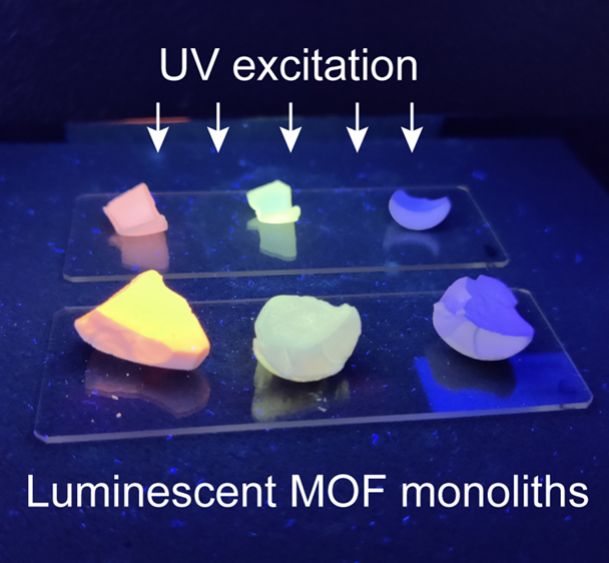

Luminescent metal–organic frameworks exhibit great potential as materials for nanophotonic applications because of their programmable properties and tunable structures. In particular, luminescent guests (LG) can be hosted by metal–organic frameworks due to their porosity and guest confinement capacity, forming LG@MOF composite systems. However, such guest–host systems are mainly produced as loose powders, preventing their widespread use in practical devices and technological applications that require implementation of a stable continuum solid. In this regard, using monolithic MOF hosts might be a workable option to solve this challenge. Herein, we reported the facile synthesis and fabrication of novel prototypical sol–gel monolithic systems, designated as LG@monoMOF. Red (rhodamine B), blue (7-methoxycoumarin), and yellow (fluorescein) emitting dyes were encapsulated in a robust UiO-66 monolithic host, resulting in the red, blue, and yellow light-emitting luminescent monoliths. The mechanical and photophysical characterization of the three LG@monoMOF systems was systematically carried out in order to unravel the role of guest–host interactions in the mechanical and optical response of the bespoke LG@monoMOF composites.

## Introduction

Metal–organic frameworks (MOFs) are nanoporous hybrid materials, whose building blocks, metal ion nodes, and organic linkers self-assemble to create a variety of lattice structures exhibiting a high internal surface area and varied physicochemical properties.

The range of potential applications of MOFs has been further expanded by the development of guest-encapsulated MOF composites, denoted as Guest@MOF.^1^ Subsequently, luminescent MOF system (LMOF) has been explored following this strategy: the porosity of MOFs can be exploited to host a variety of luminescent guests (LG), resulting in the formation of the LG@MOF systems with vastly tunable photophysical properties.^2^ A common LG is represented by organic dyes,^3,4^ metal hydroxyquinolates,^5,6^ quantum dots,^7,8^ and many other exemplars.^9−14^ Although the majority of LG materials are highly luminescent in the solution state, they experience aggregation-induced quenching when prepared in the solid state. Hitherto, this is a major impediment for the engineering of practical applications. Notably, it has been demonstrated that the confinement of LGs in the nanosized pores of MOF host can help to overcome this obstacle.^2^ To date, luminescent dyes@MOFs systems have been reported in the solid-state form as powders,^9,10^ nanoparticles,^15^ nanosheets,^6,16−18^ fibers,^19^ monoliths,^20^ etc.

One of the main reasons limiting the spread of LG@MOFs (and MOFs in general) for use in real-life devices lies in the powder morphology of the MOFs. In this sense, the use of monolithic MOF hosts could offer a viable solution.^21−24^ These structurally robust and continuous morphologies present key advantages over powders, such as easier material handling, hierarchical porosity, and functions. Monoliths also feature numerous advantages over the MOF-polymer composites,^25,26^ widely found in the literature, being the simplest method for enhancing the mechanical stability of these materials. Of note, the monolithic structure allows high effective volumetric loadings. This is in contrast to the MOF-polymer composites due to poor colloidal stability, limiting the inclusion of a high volume fraction of MOF filler causing embrittlement and cracking in a polymeric matrix.^27,28^ Moreover, the nanopores in monolithic MOFs are fully accessible,^29^ in contrast to MOF-polymer composites, where some of the internal porosity may be blocked by infiltration of polymer chains.^30^

A facile and yet effective method for producing pure MOF monoliths is by leveraging the sol–gel process, which involves the removal of solvent from a MOF gel resulting in a monolithic morphology.^21,31^ Several studies on the synthesis of sol–gel MOF monoliths have been reported in recent years for a variety of MOFs, including HKUST-1,^29,32^ ZIFs,^22,33,34^ UiO-66,^35,36^ and MIL-68.^32^ Sol–gel MOF monoliths are characterized by increased capabilities of gas uptake,^23,29,33,35^ due to different pore sizes that are found within the same hierarchical material.

Herein, we successfully synthesized and characterized three prototypical sol–gel monolithic LG@MOF systems (LG@monoMOF). The zirconium-based UiO-66 was selected as the MOF host, due to its predicted good chemical and mechanical stability.^37^ Three organic dyes were used as LGs, namely, rhodamine B (RhB), fluorescein (Fl), and 7-methoxycoumarin (7MC), corresponding to the red, yellow, and blue emitters, respectively. Both the photophysical and mechanical properties of these novel “composite” systems were studied, and the effects of guest encapsulation on the resultant structure and functions of the monoliths were explored to gain insights into the structure–property relationships.

## Experimental Section

### Materials Synthesis

Four UiO-66 reactions were started in parallel. For each batch, ZrOCl_4_·8H_2_O (2 mmol, 644 mg), terephthalic acid, and BDC (2.9 mmol, 481.4 mg) were dissolved in 10 mL of DMF. Subsequently, 1.5 mL of HCl and 2.5 mL of glacial acetic acid were added to the solution and stirred. Finally, 0.5 mL of each dye solutions (taken from a 5 mM stock) were added individually to three of the four batches. The last one was left as prepared, in order to obtain the pristine UiO-66. All samples obtained were washed 3 times with DMF and once with MeOH (centrifugation at 12,000 rpm for 10 min during each washing step). The solid products were left to dry at room temperature for 72 h.

### X-Ray Diffraction

X-ray diffraction (XRD) patterns of the monoliths were recorded by using a Rigaku MiniFlex with a Cu Kα source (1.541 Å).

### Nanoindentation Studies

An iMicro nanoindenter (KLA-Tencor), equipped with a 50 mN load cell, was used for nanoindentation testing. Cylindrical samples were produced by cold mounting the as-synthesized monoliths in an epoxy resin (Struers Epofix). The specimens were carefully polished with emery papers and then diamond suspensions (up to 0.1 μm surface finish) to minimize roughness and obtain a flat sample surface suitable for nanoindentation studies.

A Berkovich diamond indenter tip was used. Continuous stiffness measurement (CSM) technique was employed, allowing measurement of the indentation modulus (*M* obtained by letting the Poisson’s ratio of sample, ν_s_ = 0)^38^ and hardness (*H*) as a function of the surface penetration depth. The maximum indentation depth was set to 2000 nm for all of the tests. Thermal drift was determined at 90% unload for a period of 60 s. Average values of *M* and *H* were computed from the surface penetration depths of 500–2000 nm. The NanoBlitz 3D technique was also employed to generate indentation maps of local mechanical properties across the sample surface of 200 μm × 100 μm.

### Density Measurements

The density of the monoliths was determined using Archimedes’ principle by measuring its weight in air and when submerged in an auxiliary liquid (distilled water). A Mettler Toledo laboratory balance equipped with a density kit was employed.

### Solution ^1^H Nuclear Magnetic Resonance (NMR) Spectroscopy

Monolithic samples were ground to a fine powder, and then, 15–20 mg was dissolved in a solution composed of 500 μL of methanol-d4 and 50 μL of DCl/D_2_O (35 wt %). All ^1^H NMR spectroscopy of the acid-digested samples was collected at 298 K using a Bruker Avance spectrometer operating at 600 MHz, equipped with a BBO cryoprobe. Data were collected using a relaxation delay of 20 s, with 128,000 points and a sweep width of 19.8 ppm, giving a digital resolution of 0.18 Hz. Data were processed using Bruker Topspin with a line broadening of 1 Hz and 2 rounds of zero-filling.

### ATR-FTIR

Attenuated total reflection Fourier transform infrared (ATR-FTIR) spectra were recorded by using a Nicolet iS10 FTIR spectrometer, employing a diamond crystal mounted on the ATR module.

### Nearfield Infrared Nanospectroscopy (nano-FTIR)

Nanoscale Fourier transform infrared (nano-FTIR) spectroscopy^39^ was performed using a neaSNOM instrument (Neaspec GmbH), where a platinum-coated atomic force microscope (AFM) probe (Arrow-NCPt, tip radius <25 nm, 285 kHz) under the tapping mode is illuminated by a broadband mid-IR difference frequency generation (DFG) laser source (Toptica). Local nano-FTIR spectra were acquired in the two phases of each sample. Each nano-FTIR point spectrum was derived from nearfield nanospectroscopy of an average of 10 individual interferograms taken on the same spot (probe size ∼20 nm), with 1024 pixels and an integration time of 11 ms per pixel, normalized by a reference spectrum taken on a silicon wafer. AFM height topography images were also determined under the tapping mode.

### Photophysical Properties Characterization

Solid-state photoluminescence (PL) spectra were recorded by using the FS-5 spectrofluorometer (Edinburgh Instruments). The spectrofluorometer equipped with the SC-10 module facilitated the acquisition of steady-state excitation–emission spectra as well as time-correlated single-photon counting (TCSPC) emission decay data of solid-state samples. For the TCSPC measurements of the emission lifetime, a 365 nm EPLED picosecond pulsed laser source was utilized to excite the samples. Photoluminescent quantum yield (PLQY) measurements were conducted by using the SC-30 module. Data analysis was performed by using the Fluoracle software. It is worth noting that all emission lifetime and quantum yield measurements were performed using mm thick monoliths of ca. 1 × 1 cm^2^ area, as dictated by the default size of the sample holder in the FS-5. As such, the size-dependent attenuation effects were not significantly varied across the monoliths being tested in this study.

## Results and Discussion

**Figure 1:**
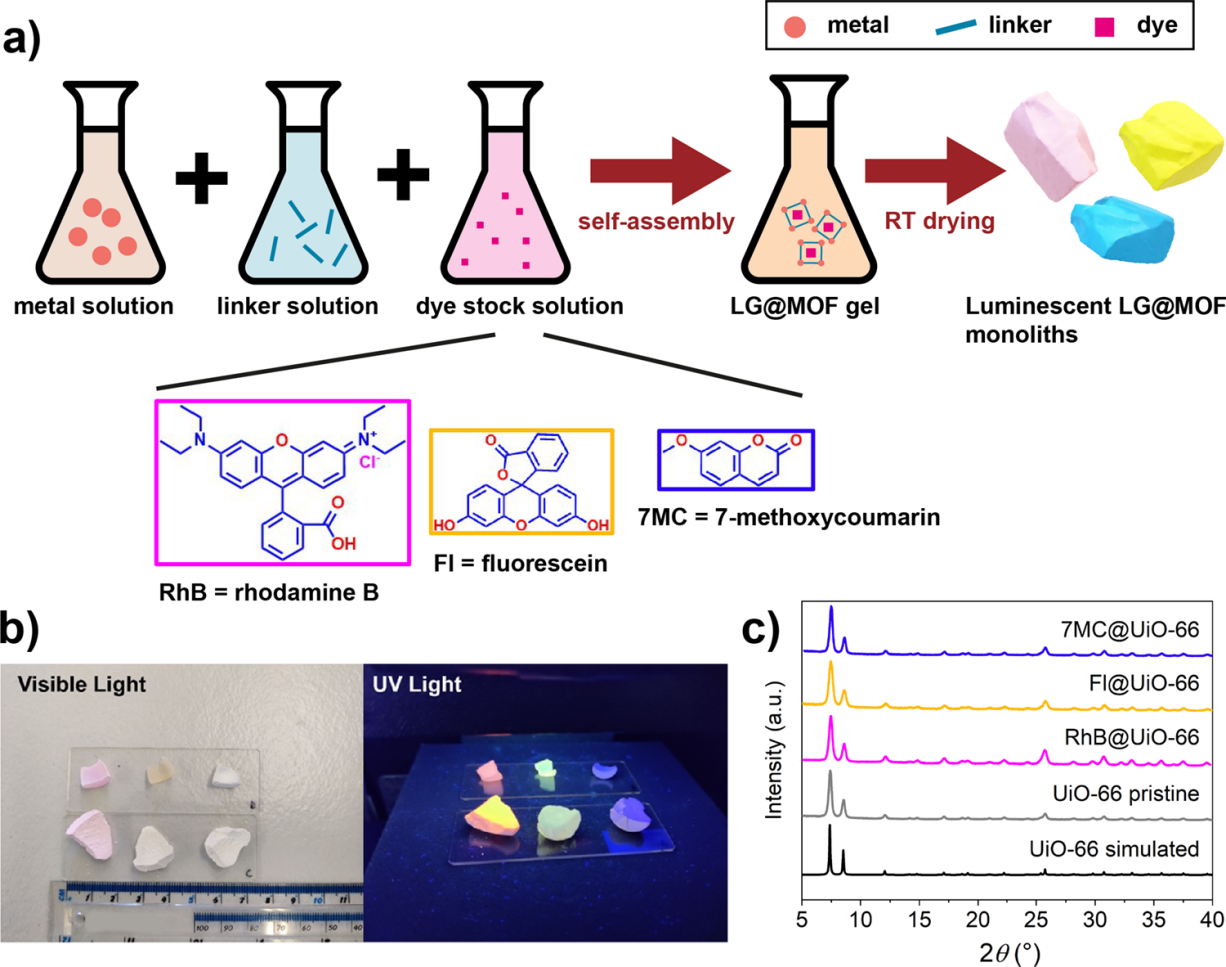
(a) Schematic of the synthesis route used to fabricate the LG@MOF monoliths. (b) Optical images of the monoliths under visible light and 365 nm UV light. (c) XRD patterns of pristine UiO-66 monolith and the three LG@monoUiO-66 systems.

The three LG@monoMOF composites were fabricated via a single-step in situ guest confinement strategy (Figure 1a). A detailed description of the synthesis protocol is given in the Methods section. Guided by our previous study,^3^ the guest concentration used during the synthesis was set to 5 mM. We obtained cm-sized monoliths that luminesce under UV light (Figure 1b). XRD patterns confirmed the successful synthesis of UiO-66 in all cases (Figure 1c).

**Figure 2:**
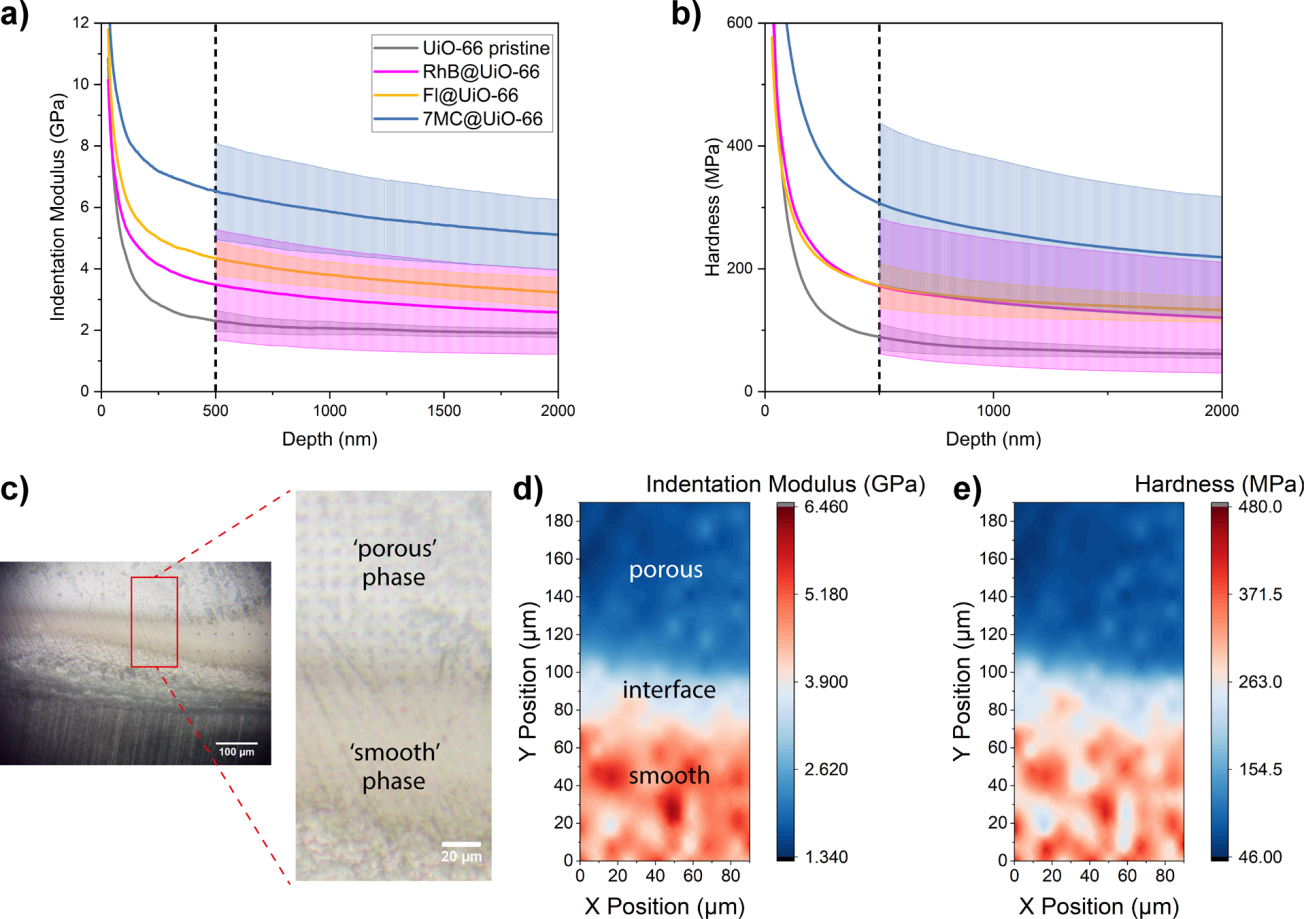
Nanoindentation of the LG@monoUiO-66 composites. Averaged (a) indentation modulus and (b) hardness vs indentation depth, resulting from the CSM nanoindentation tests (see Methods). (c) Optical micrograph of the surface of the RhB@UiO-66 sample, with a magnified view of the area on which NanoBlitz 3D mapping (see Methods) was performed. (d) Indentation modulus and (e) hardness maps of the area highlighted in (c).

**Figure 3:**
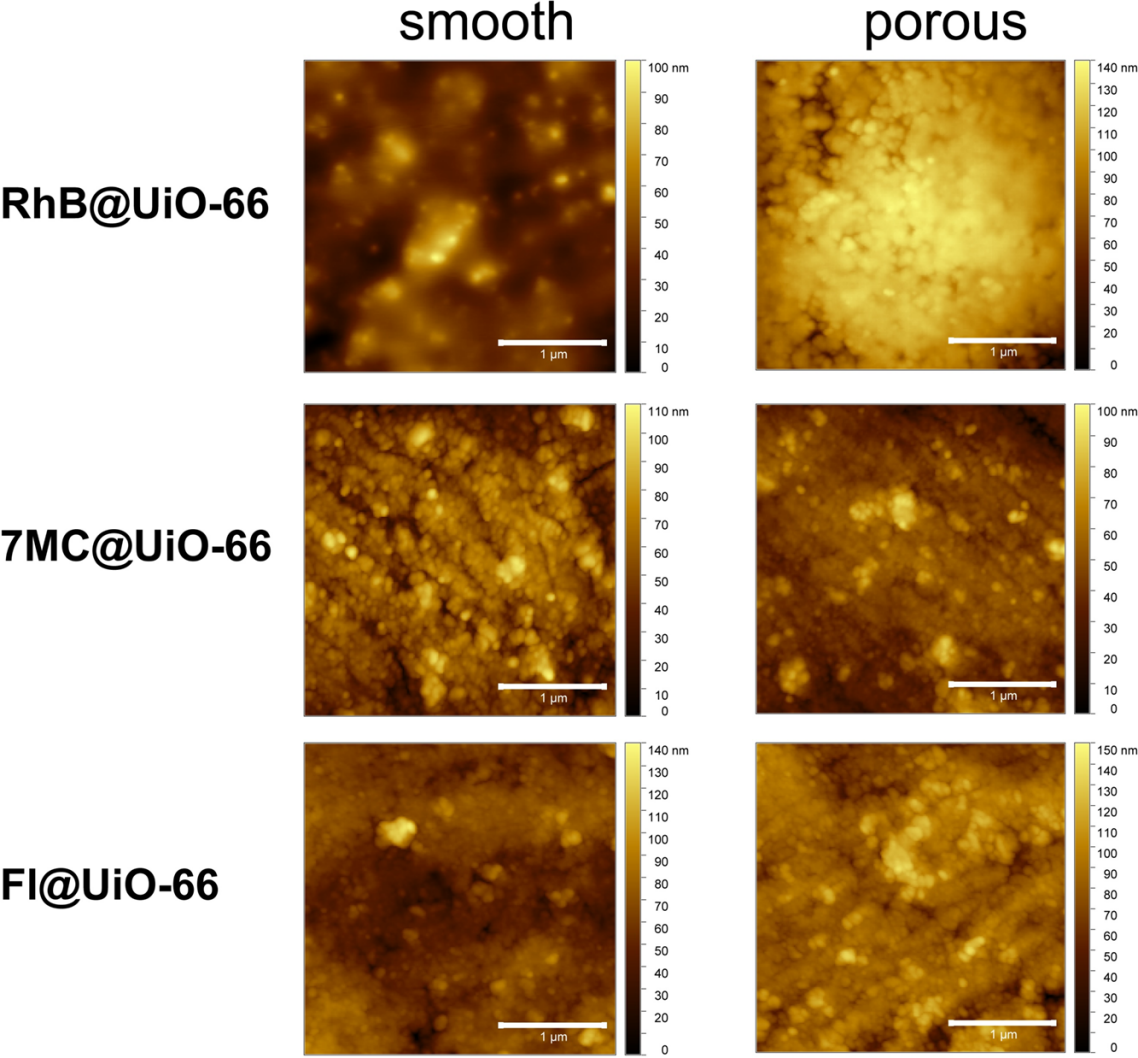
AFM height topography of the LG@MOF monoliths reveals the local regions that exhibit differential surface porosity and nanoscale defects.

Nanoindentation tests were carried out to study the mechanical response of the composites. As shown in Figure 2 and Table 1, guest encapsulation improved both the indentation modulus (*M*) and hardness (*H*) of the pristine UiO-66 monolith with 7MC@UiO-66 outperforming the RhB and Fl counterparts. However, these results exhibit a significant scatter, quantified by the large standard deviation of the indentation modulus and hardness values. This can be explained by the presence of two different “phases” within the materials, which exhibit different optical and mechanical properties (Figures S1 and S2). As an example, a cross-section of RhB@UiO-66 is shown in Figure 2c, for which a “smooth” and a “porous” phase can be clearly told apart. We performed the micromechanical mapping by employing the NanoBlitz method implemented in a KLA iMicro nanoindenter (see Methods). The resulting contour maps of the indentation modulus (Figure 2d) and hardness (Figure 2e) clearly demonstrate the presence of two different phases: the “smooth” phase is both stiffer and harder than the “porous” counterpart. Based on the nanomechanical finding, we propose that these two phases were derived from a nonhomogenous aggregation of the MOF nanoparticles constituting the monoliths. Figure 3 shows the AFM scans of the local regions present on the LG@MOF monoliths, revealing the nanostructured morphology of the consolidated nanoparticles found on the surface. Notably, the topographic images reveal the fine-scale porosity and surface defects evidenced on the surface of the monoliths.

**Table 1:** Mechanical Properties and Density (Average of 3 Measurements) of the UiO-66-Based Monoliths^a^

monolith sample	number of nanoindentation tests	indentation modulus, *M* (GPa)	hardness, *H* (MPa)	density (g cm^–3^)
UiO-66 pristine^b^	32	2.06 ± 0.20	72 ± 12	1.348 ± 0.011
RhB@UiO-66	33	3.02 ± 1.59	145 ± 101	1.42 ± 0.005
Fl@UiO-66	28	3.79 ± 0.42	151 ± 27	1.270 ± 0.001
7MC@UiO-66	43	5.91 ± 1.38	263 ± 114	1.316 ± 0.011

a Nanoindentation measurements were performed at a maximum surface penetration depth of 2000 nm.

b Note the larger scatter and differential values of elastic modulus and hardness compared with the previous report by Connolly et al.^23^ These differences could be attributed to the variation in the synthetic and drying protocols employed to yield MOF monoliths.

**Figure 4:**
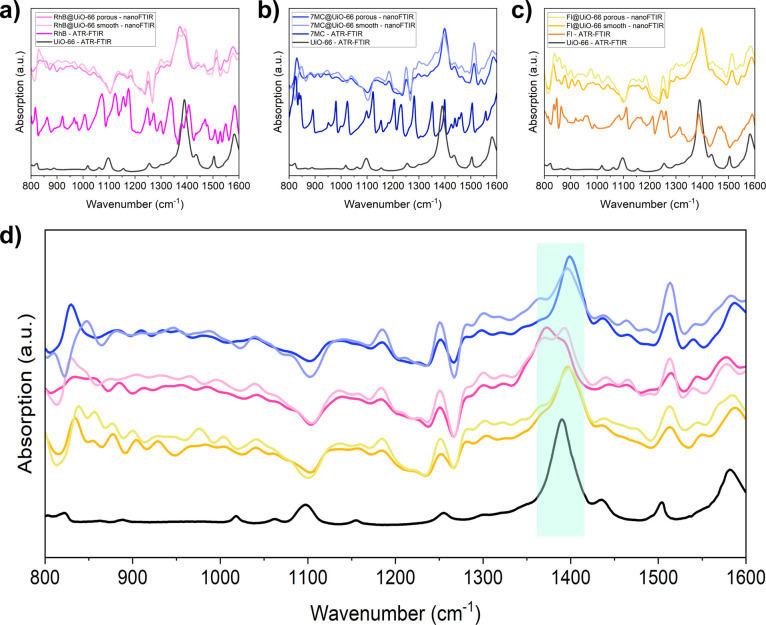
Far-field ATR-FTIR and nearfield nano-FTIR spectra of (a) RhB@monoUiO-66, (b) 7MC@monoUiO-66, and (c) Fl@monoUiO-66. (d) Comparison of the nano-FTIR (local) spectra of the porous and smooth phase of the three composites and the ATR-FTIR (bulk) spectrum of the pristine UiO-66 monolith (black trace). Characteristic vibrational mode of UiO-66 at 1390 cm^–1^ is highlighted in green.

Such a hypothesis was confirmed by nearfield characterization via nano-Fourier transform infrared (nano-FTIR) spectroscopy: local IR absorbance spectra were collected on the two phases for each sample. No major differences were detected in the characteristic spectra of UiO-66, thereby revealing that the chemical structures of the two phases are similar (Figure 4d). The nano-FTIR “local” spectra of the two phases also match the ATR-FTIR “bulk” spectrum of the UiO-66 sample (black in Figure 4), suggesting that the framework structure was preserved upon guest encapsulation. Bulk ATR-FTIR spectra of the composites (Figures 4a–c and S3) were also collected and compared with those of the pristine dyes and UiO-66. As expected, the spectra of the composites matched the pristine UiO-66 spectrum, given the low concentration of the guest loading used; this notion is supported by solution ^1^H NMR spectroscopy presented in Figures S4–S6. For example, we estimated that the guest concentration of the RhB@UiO-66 monolith is 1 rhodamine B for every 86.2 pores of UiO-66, while it is relatively lower for the Fl@UiO-66 monolith with 1 fluorescein for every 400 pores.

The only difference detectable from nano-FTIR spectra of the two phases is a slight merging of the main characteristic peak of UiO-66 at ∼1390 cm^–1^ with the peaks of the RhB dye, which might suggest that the “porous” phase is richer in guest, probably in an aggregated form, sitting inside intergranular macropores. The presence of intergranular macropores would justify the relatively lower mechanical stiffness and hardness determined for the “porous” phase. On the other hand, the higher stiffness and hardness of the “smooth” phase might be due to the dyes well confined as guests within the nanopores of the UiO-66 host. This speculation could also explain why no changes whatsoever are detected in the nano-FTIR spectra of the Fl@UiO-66 and 7MC@UiO-66 composites: Fl and 7MC molecules are in fact relatively smaller than RhB (see Figure 1a) (176 and 332 g/mol against 479 g/mol, respectively) and can more easily fit into the nanoscale pores of the UiO-66 MOF host.

The absence of aggregated dyes on the surface of the monolith is confirmed by the absence of characteristic vibrational peaks of the guests in the nano-FTIR spectra, in line with the results of Möslein et al.^40^

**Figure 5:**
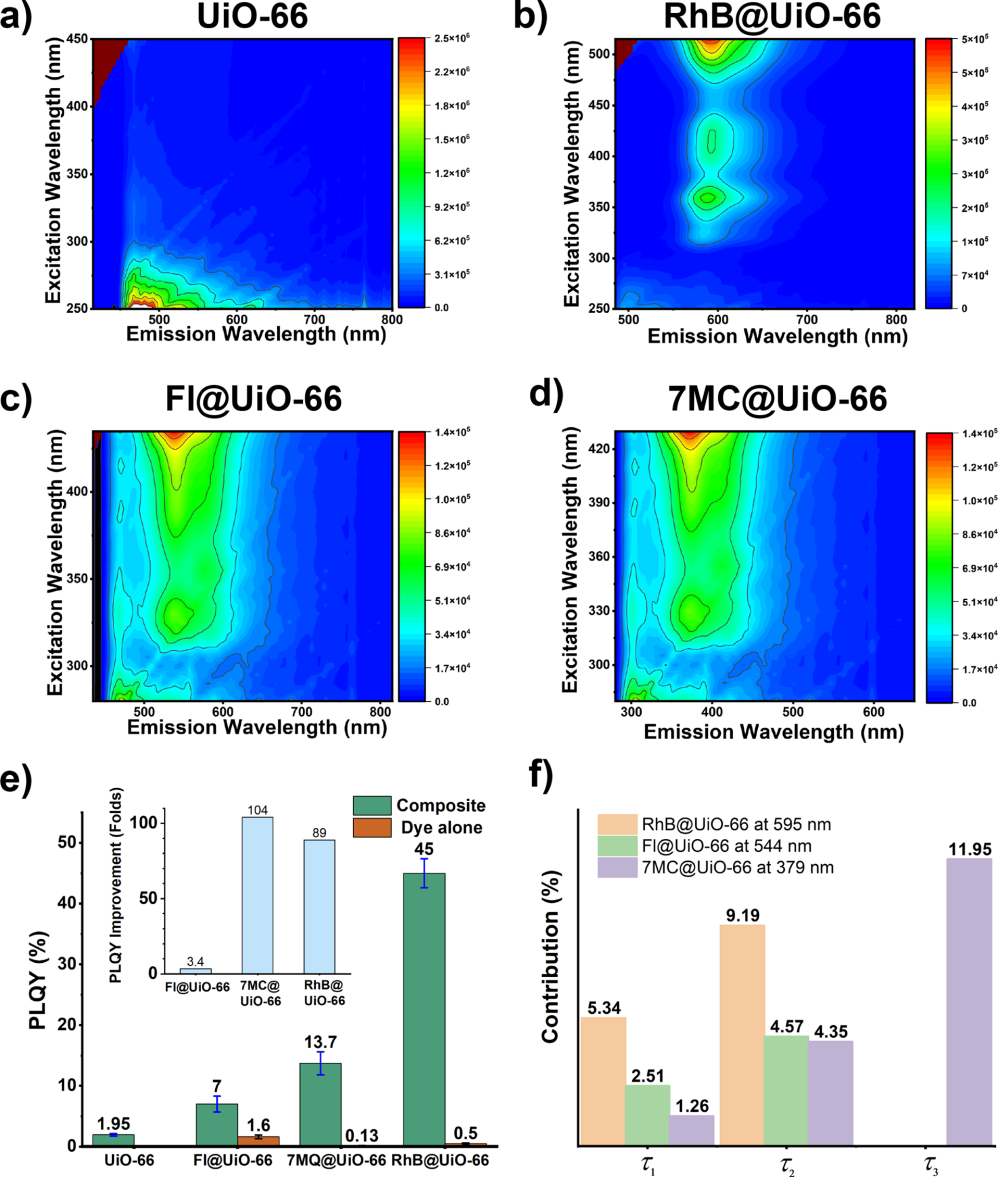
Photoluminescent properties. (a–d) Excitation–emission maps of pristine monoUiO-66 and the three LG@monoUiO-66 composites. (e) PLQY of pristine UiO-66, the three LG@monoUiO-66 composites, and the pristine dyes. Inset shows the relative improvement of the composites compared with those of the respective pristine dyes. (f) Time constants derived from fluorescence decay lifetime measurements by the TCSPC technique at emission maximum. τ_1_ = H-aggregates, τ_2_ = J-aggregates, and τ_3_ = monomer species (Table S1).

The detailed photophysics of luminescent monoliths presented in this work reveal intriguing insights into their luminescence behavior. Our study covers the entire visible color spectrum, achieving the blue, green, and yellowish orange emissions by encapsulating corresponding fluorophores such as 7MC, Fl, and RhB within the UiO-66 host. Initially, the excitation and emission peaks of each monolith were determined (Figures S7 and S8). The blue-emitting monolith (7MC@UiO-66) exhibited excitation and emission maxima at 325 and 370 nm, respectively. The monolith labeled as Fl@UiO-66 emitted green light with excitation and emission peaks at 334 and 550 nm, respectively. On the other hand, the monolith labeled as RhB@UiO-66 emitted yellow–orange light with excitation and emission peaks located at 356 and 590 nm, respectively (Figure 5a–d).

The solid-state photoluminescent quantum yield (PLQY) of these monoliths was found to be remarkably high. Specifically, the *QY* values for the blue, green, and yellow-orange light-emitting monoliths were determined as 7% ± 1.3, 13.7% ± 1.9, and 45% ± 2.4, respectively. In comparison, the pristine UiO-66 monoliths had a *QY* of only 1.9% ± 0.4. These values significantly surpass the *QY* values of pristine dye molecules (Figure 5e).

Furthermore, our analysis delved deeper into photophysics through time-correlated single-photon counting (TCSPC) measurements. The decay curves (Figure S9) exhibit multiple lifetime τ components (Figure 5f) suggesting the coexistence of different molecular species with varying lifetimes, indicating a mixture of monomeric and aggregated forms of dye molecules in the monoliths. For instance, the yellow–orange emitting monoliths exhibited a lifetime component greater than 7–8 ns, indicating the presence of monomers, while a component of 5.34 ns points to a possibility of dye aggregation on the surface.^41^ Similarly, the green-emitting monoliths showed lifetime components of τ_1_ = 2.51 ns and τ_2_ = 4.57 ns, suggesting the presence of predominantly the ‘J’-type aggregated forms of the fluorescein dye within the monolithic materials.^3^ In contrast, the blue-emitting monoliths displayed a lifetime component of τ_1_ = 1.26 ns, indicative of monomeric states, and a lifetime component of τ_3_ = 11.95 ns, signifying aggregation of the 7MC dye molecules. The presence of an intermediate lifetime component of τ_2_ = 4.35 ns indicates the coexistence of monomeric and excimer states within the blue-emitting monolithic materials.^42,43^ Since the majority of the molecules existed in aggregated forms, the overall *QY* improvements in Fl@UiO-66 monolithic materials are quite low compared to those of the 7MC@UiO-66 and RhB@UiO-66 monoliths.

## Conclusions

In summary, we have successfully fabricated novel LG@MOF monoliths by leveraging a facile sol–gel synthesis route. Mechanical, chemical, and optical characterization have been systematically performed, in an effort to relate photophysical and mechanical behavior of the luminescent composite monoliths.

The mechanical properties of the three composites outperformed those of pristine host UiO-66, indicating an effect of the organic luminescent dyes as guests on the monolith structure. Particularly, two phases were distinguished in the composites (i.e., a “porous” phase and a “smooth” phase), which exhibited similar chemical properties (as demonstrated by local nano-FTIR spectra), but exhibit differential mechanical behavior in terms of elastic stiffness and hardness. We hypothesize that such a phase separation was due to the nonhomogenous aggregation of MOF nanoparticles forming the monoliths, resulting from dye encapsulation. The thorough investigation of photophysics shows that the aggregated forms of fluorescein dye are predominantly present in the monoliths. This is reinforced by the notably reduced quantum yield of green-emitting Fl@UiO-66 monoliths compared with other monoliths investigated in this study. Overall, this first study on the intricate photophysics and mechanics of luminescent monoliths has provided valuable insights into their molecular states in the “bulk” MOF solids, paving the way for their potential applications in multifunctional sensing devices and optoelectronics.

## Supporting Information Available

Optical microscopy images, nanoindentation load–displacement curves, ATR-FTIR vibrational spectra, NMR spectra and guest loading, photoluminescent excitation–emission spectra, TCSPC lifetime emission spectra, and fluorescence decay lifetimes (PDF)

## References

[ref1] Allendorf M. D.; Foster M. E.; Leonard F.; Stavila V.; Feng P. L.; Doty F. P.; Leong K.; Ma E. Y.; Johnston S. R.; Talin A. A. Guest-induced emergent properties in metal-organic frameworks. J. Phys. Chem. Lett. 2015, 6, 1182–1195. 10.1021/jz5026883. 26262970

[ref2] Gutiérrez M.; Zhang Y.; Tan J. C. Confinement of luminescent guests in metal-organic frameworks: Understanding pathways from synthesis and multimodal characterization to potential applications of LG@MOF systems. Chem. Rev. 2022, 122, 10438–10483. 10.1021/acs.chemrev.1c00980. 35427119 PMC9185685

[ref3] Xiong T.; Zhang Y.; Donà L.; Gutiérrez M.; Möslein A. F.; Babal A. S.; Amin N.; Civalleri B.; Tan J. C. Tunable fluorescein-encapsulated zeolitic imidazolate framework-8 nanoparticles for solid-state lighting. ACS Appl. Nano Mater. 2021, 4, 10321–10333. 10.1021/acsanm.1c01829.

[ref4] Zhang D. S.; Gao Q.; Chang Z.; Liu X. T.; Zhao B.; Xuan Z. H.; Hu T. L.; Zhang Y. H.; Zhu J.; Bu X. H. Rational construction of highly tunable donor-acceptor materials based on a crystalline host-guest platform. Adv. Mater. 2018, 30, e1804715 10.1002/adma.201804715. 30318756

[ref5] Mollick S.; Rai S.; Frentzel-Beyme L.; Kachwal V.; Donà L.; Schürmann D.; Civalleri B.; Henke S.; Tan J. C. Unlocking diabetic acetone vapor detection by a portable metal-organic framework-based turn-on optical sensor device. Adv. Sci. 2024, 11, 2305070 10.1002/advs.202305070. PMC10811499 38032122

[ref6] Chaudhari A. K.; Kim H. J.; Han I.; Tan J. C. Optochemically responsive 2D nanosheets of a 3D metal-organic framework material. Adv. Mater. 2017, 29, 1701463 10.1002/adma.201701463. 28488776

[ref7] Asadi F.; Azizi S. N.; Chaichi M. J. Green synthesis of fluorescent PEG-ZnS QDs encapsulated into Co-MOFs as an effective sensor for ultrasensitive detection of copper ions in tap water. Mater. Sci. Eng., C 2019, 105, 110058 10.1016/j.msec.2019.110058. 31546432

[ref8] Sun J. N.; Zhang P.; Yan K.; Pan A. Z.; Chen F.; Hong J.; Zhao C. Y.; Chen X. H.; Xiong W. Europium/1,3,5-benzenetricarboxylic acid metal-organic framework nanorods decorated with CdSe quantum dots as coatings for noncontact ratiometric optical temperature sensing. ACS Appl. Nano Mater. 2023, 6, 12087–12094. 10.1021/acsanm.3c01852.

[ref9] Lustig W. P.; Mukherjee S.; Rudd N. D.; Desai A. V.; Li J.; Ghosh S. K. Metal-organic frameworks: Functional luminescent and photonic materials for sensing applications. Chem. Soc. Rev. 2017, 46, 3242–3285. 10.1039/C6CS00930A. 28462954

[ref10] Leith G. A.; Martin C. R.; Mayers J. M.; Kittikhunnatham P.; Larsen R. W.; Shustova N. B. Confinement-guided photophysics in MOFs, COFs, and cages. Chem. Soc. Rev. 2021, 50, 4382–4410. 10.1039/D0CS01519A. 33594994

[ref11] Mollick S.; Zhang Y.; Kamal W.; Tricarico M.; Möslein A. F.; Kachwal V.; Amin N.; Castrejón-Pita A. A.; Morris S. M.; Tan J. C. Resilient photoswitchable metal-organic frameworks for sunlight-induced on-demand photochromism in the solid state. Chem. Eng. J. 2023, 476, 146727 10.1016/j.cej.2023.146727.

[ref12] Zhang M.; Feng G. X.; Song Z. G.; Zhou Y. P.; Chao H. Y.; Yuan D. Q.; Tan T. T. Y.; Guo Z. G.; Hu Z. G.; Tang B. Z.; et al. Two-dimensional metal-organic framework with wide channels and responsive turn-on fluorescence for the chemical sensing of volatile organic compounds. J. Am. Chem. Soc. 2014, 136, 7241–7244. 10.1021/ja502643p. 24824627

[ref13] Kaur H.; Sundriyal S.; Pachauri V.; Ingebrandt S.; Kim K. H.; Sharma A. L.; Deep A. Luminescent metal-organic frameworks and their composites: Potential future materials for organic light emitting displays. Coord. Chem. Rev. 2019, 401, 213077 10.1016/j.ccr.2019.213077.

[ref14] Cui Y. J.; Yue Y. F.; Qian G. D.; Chen B. L. Luminescent functional metal-organic frameworks. Chem. Rev. 2012, 112, 1126–1162. 10.1021/cr200101d. 21688849

[ref15] Cha J. H.; Noh K.; Yin W.; Lee Y.; Park Y.; Ahn T. K.; Mayoral A.; Kim J.; Jung D. Y.; Terasaki O. Formation and encapsulation of all-inorganic lead halide perovskites at room temperature in metal-organic frameworks. J. Phys. Chem. Lett. 2019, 10, 2270–2277. 10.1021/acs.jpclett.9b00510. 31002525

[ref16] Sherman D. A.; Gutiérrez M.; Griffiths I.; Mollick S.; Amin N.; Douhal A.; Tan J. C. Guest entrapment in metal-organic nanosheets for quantifiably tuneable luminescence. Adv. Funct. Mater. 2023, 33, 2214307 10.1002/adfm.202214307.

[ref17] Huang M. Y.; Liang Z. X.; Huang J. L.; Wen Y. H.; Zhu Q. L.; Wu X. T. Introduction of multicomponent dyes into 2D MOFs: A strategy to fabricate white light-emitting MOF composite nanosheets. ACS Appl. Mater. Interfaces 2023, 15, 11131–11140. 10.1021/acsami.2c22568. 36799618

[ref18] Tian R.; Zhang S. T.; Li M. W.; Zhou Y. Q.; Lu B.; Yan D. P.; Wei M.; Evans D. G.; Duan X. Localization of Au nanoclusters on layered double hydroxides nanosheets: Confinement-induced emission enhancement and temperature-responsive luminescence. Adv. Funct. Mater. 2015, 25, 5006–5015. 10.1002/adfm.201501433.

[ref19] Kachwal V.; Mollick S.; Tan J. C. Tailored broad-spectrum emission in hybrid aggregation induced emission (AIE)-MOFs: Boosting white light efficiency in electrospun Janus microfibers. Adv. Funct. Mater. 2024, 34, 2308062 10.1002/adfm.202308062.

[ref20] Mollick S.; Mandal T. N.; Jana A.; Fajal S.; Ghosh S. K. A hybrid blue perovskite@metal-organic gel (MOG) nanocomposite: Simultaneous improvement of luminescence and stability. Chem. Sci. 2019, 10, 10524–10530. 10.1039/C9SC03829A. 32110340 PMC7020792

[ref21] Hou J.; Sapnik A. F.; Bennett T. D. Metal–organic framework gels and monoliths. Chem. Sci. 2020, 11, 310–323. 10.1039/C9SC04961D. 32153752 PMC7021205

[ref22] Tricarico M.; Tan J. C. Mechanical properties and nanostructure of monolithic zeolitic imidazolate frameworks: A nanoindentation, nanospectroscopy, and finite element study. Mater. Today Nano 2022, 17, 100166 10.1016/j.mtnano.2021.100166.

[ref23] Connolly B. M.; Madden D. G.; Wheatley A. E. H.; Fairen-Jimenez D. Shaping the future of fuel: Monolithic metal-organic frameworks for high-density gas storage. J. Am. Chem. Soc. 2020, 142, 8541–8549. 10.1021/jacs.0c00270. 32294384

[ref24] Lim G. J. H.; Wu Y.; Shah B. B.; Koh J. J.; Liu C. K.; Zhao D.; Cheetham A. K.; Wang J.; Ding J. 3D-printing of pure metal-organic framework monoliths. ACS Mater. Lett. 2019, 1, 147–153. 10.1021/acsmaterialslett.9b00069.

[ref25] Kalaj M.; Bentz K. C.; Ayala S. Jr.; Palomba J. M.; Barcus K. S.; Katayama Y.; Cohen S. M. MOF-polymer hybrid materials: From simple composites to tailored architectures. Chem. Rev. 2020, 120, 8267–8302. 10.1021/acs.chemrev.9b00575. 31895556

[ref26] Dong G. X.; Li H. Y.; Chen V. K. Challenges and opportunities for mixed-matrix membranes for gas separation. J. Mater. Chem. A 2013, 1, 4610–4630. 10.1039/c3ta00927k.

[ref27] Smith S. J. D.; Lau C. H.; Mardel J. I.; Kitchin M.; Konstas K.; Ladewig B. P.; Hill M. R. Physical aging in glassy mixed matrix membranes; tuning particle interaction for mechanically robust nanocomposite films. J. Mater. Chem. A 2016, 4, 10627–10634. 10.1039/C6TA02603F.

[ref28] Mahdi E. M.; Tan J. C. Mixed-matrix membranes of zeolitic imidazolate framework (ZIF-8)/Matrimid nanocomposite: Thermo-mechanical stability and viscoelasticity underpinning membrane separation performance. J. Membr. Sci. 2016, 498, 276–290. 10.1016/j.memsci.2015.09.066.

[ref29] Tian T.; Zeng Z.; Vulpe D.; Casco M. E.; Divitini G.; Midgley P. A.; Silvestre-Albero J.; Tan J. C.; Moghadam P. Z.; Fairen-Jimenez D. A sol-gel monolithic metal-organic framework with enhanced methane uptake. Nat. Mater. 2018, 17, 174–179. 10.1038/nmat5050. 29251723

[ref30] Semino R.; Moreton J. C.; Ramsahye N. A.; Cohen S. M.; Maurin G. Understanding the origins of metal-organic framework/polymer compatibility. Chem. Sci. 2018, 9, 315–324. 10.1039/C7SC04152G. 29629100 PMC5868319

[ref31] Hou X.; Sun J.; Lian M.; Peng Y.; Jiang D.; Xu M.; Li B.; Xu Q. Emerging synthetic methods and applications of MOF-based gels in supercapacitors, water treatment, catalysis, adsorption, and energy storage. Macromol. Mater. Eng. 2022, 308, 2200469 10.1002/mame.202200469.

[ref32] Tricarico M.; Tan J. C. Nanostructure-dependent indentation fracture toughness of metal-organic framework monoliths. Next Mater. 2023, 1, 100009 10.1016/j.nxmate.2023.100009.

[ref33] Tian T.; Velazquez-Garcia J.; Bennett T. D.; Fairen-Jimenez D. Mechanically and chemically robust ZIF-8 monoliths with high volumetric adsorption capacity. J. Mater. Chem. A 2015, 3, 2999–3005. 10.1039/C4TA05116E.

[ref34] Hunter-Sellars E.; Saenz-Cavazos P. A.; Houghton A. R.; McIntyre S. R.; Parkin I. P.; Williams D. R. Sol–gel synthesis of high-density zeolitic imidazolate framework monoliths via ligand assisted methods: Exceptional porosity, hydrophobicity, and applications in vapor adsorption. Adv. Funct. Mater. 2020, 31, 2008357 10.1002/adfm.202008357.

[ref35] Connolly B. M.; Aragones-Anglada M.; Gandara-Loe J.; Danaf N. A.; Lamb D. C.; Mehta J. P.; Vulpe D.; Wuttke S.; Silvestre-Albero J.; Moghadam P. Z.; et al. Tuning porosity in macroscopic monolithic metal-organic frameworks for exceptional natural gas storage. Nat. Commun. 2019, 10, 2345 10.1038/s41467-019-10185-1. 31138802 PMC6538620

[ref36] Bueken B.; Van Velthoven N.; Willhammar T.; Stassin T.; Stassen I.; Keen D. A.; Baron G. V.; Denayer J. F. M.; Ameloot R.; Bals S.; et al. Gel-based morphological design of zirconium metal-organic frameworks. Chem. Sci. 2017, 8, 3939–3948. 10.1039/C6SC05602D. 28553536 PMC5433495

[ref37] Wu H.; Yildirim T.; Zhou W. Exceptional mechanical stability of highly porous zirconium metal-organic framework UiO-66 and its important implications. J. Phys. Chem. Lett. 2013, 4, 925–930. 10.1021/jz4002345. 26291357

[ref38] Tan J. C. Fundamentals of MOF mechanics & structure–mechanical property relationships. In Mechanical behaviour of metal – organic framework materials; Tan J. C., Ed.; Royal Society of Chemistry, 2023; pp 1–64.

[ref39] Mester L.; Govyadinov A. A.; Chen S.; Goikoetxea M.; Hillenbrand R. Subsurface chemical nanoidentification by nano-FTIR spectroscopy. Nat. Commun. 2020, 11, 3359 10.1038/s41467-020-17034-6. 32620874 PMC7335173

[ref40] Moslein A. F.; Gutierrez M.; Cohen B.; Tan J. C. Near-field infrared nanospectroscopy reveals guest confinement in metal-organic framework single crystals. Nano Lett. 2020, 20, 7446–7454. 10.1021/acs.nanolett.0c02839. 32870694

[ref41] Xiong T.; Zhang Y.; Amin N.; Tan J. C. A luminescent guest@MOF nanoconfined composite system for solid-state lighting. Molecules 2021, 26, 7583 10.3390/molecules26247583. 34946662 PMC8706567

[ref42] Park S.-Y.; Ebihara M.; Kubota Y.; Funabiki K.; Matsui M. The relationship between solid-state fluorescence intensity and molecular packing of coumarin dyes. Dyes Pigm. 2009, 82, 258–267. 10.1016/j.dyepig.2009.01.014.

[ref43] Wondraczek H.; Kotiaho A.; Niemi M.; Fardim P.; Heinze T. Studies on the structure of coumarin-modified dextran nanoparticles by fluorescence spectroscopy. Carbohydr. Polym. 2013, 97, 45–51. 10.1016/j.carbpol.2013.04.040. 23769515

