# Supplementary material for: Exploring the Photophysical and Mechanical Behavior of Fluorescent Metal–Organic Framework Monoliths
Source: Chem Mater. 2024 Aug 20;36(17):8247–54. doi: 10.1021/acs.chemmater.4c00963 (PMC11393794; doi:10.1021/acs.chemmater.4c00963)
Supplement: Supplementary file 1 — cm4c00963_si_001.pdf [file cm4c00963_si_001.pdf]

Supporting Information

for

Exploring the Photophysical and Mechanical Behavior of Fluorescent Metal-Organic Framework Monoliths

Michele Tricarico, Samraj Mollick, Vishal Kachwal, Dylan A. Sherman and Jin-Chong Tan*

Multifunctional Materials and Composites (MMC) Laboratory, Department of Engineering Science, University of Oxford, Parks Road, Oxford, OX1 3PJ, United Kingdom.

*Email: jin-chong.tan@eng.ox.ac.uk

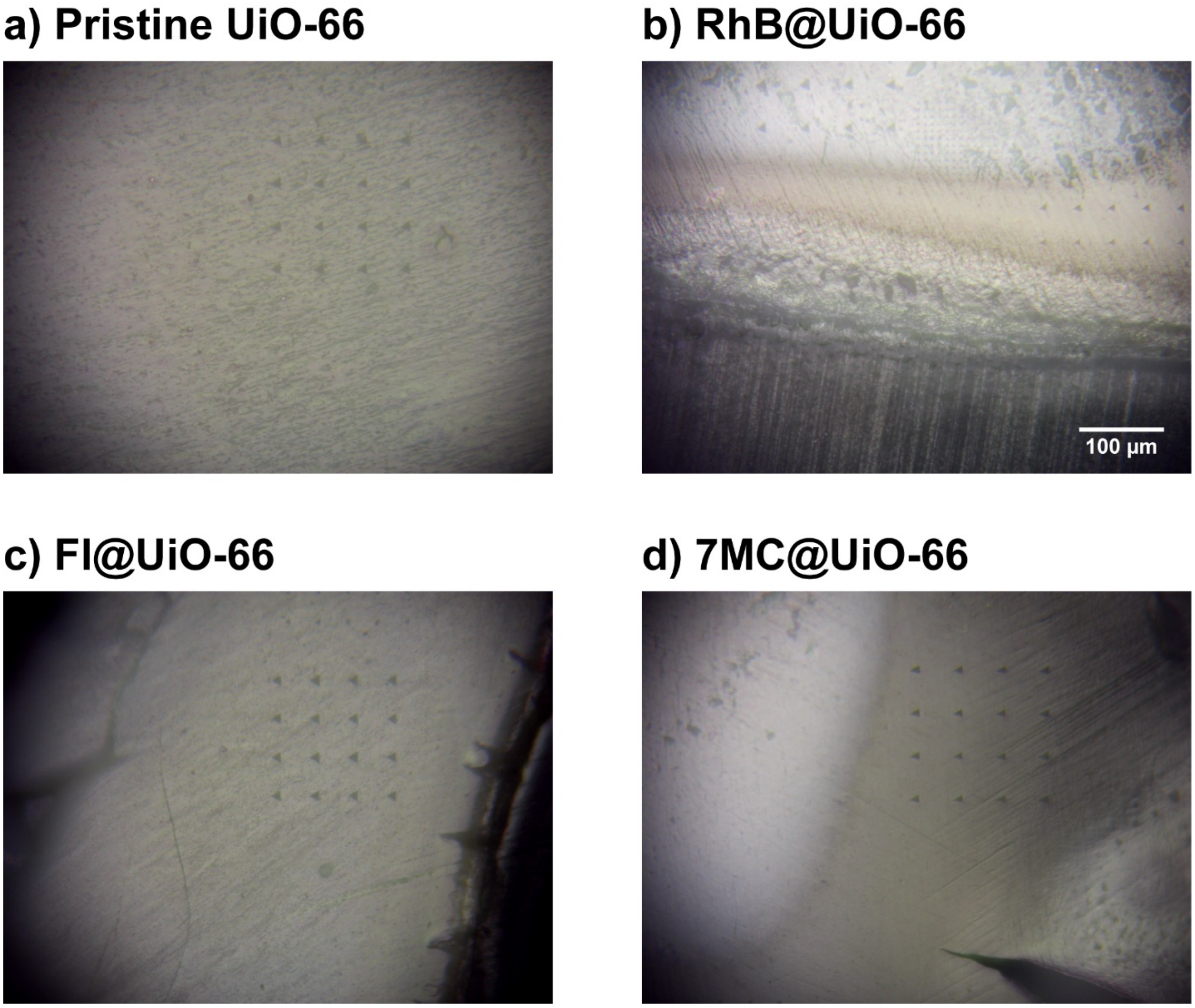

Figure S1: Cross sections of the composites samples of UiO-66 and LG@monoUiO-66 employed in the nanoindentation studies. The presence of two phases in the composites is clearly visible.

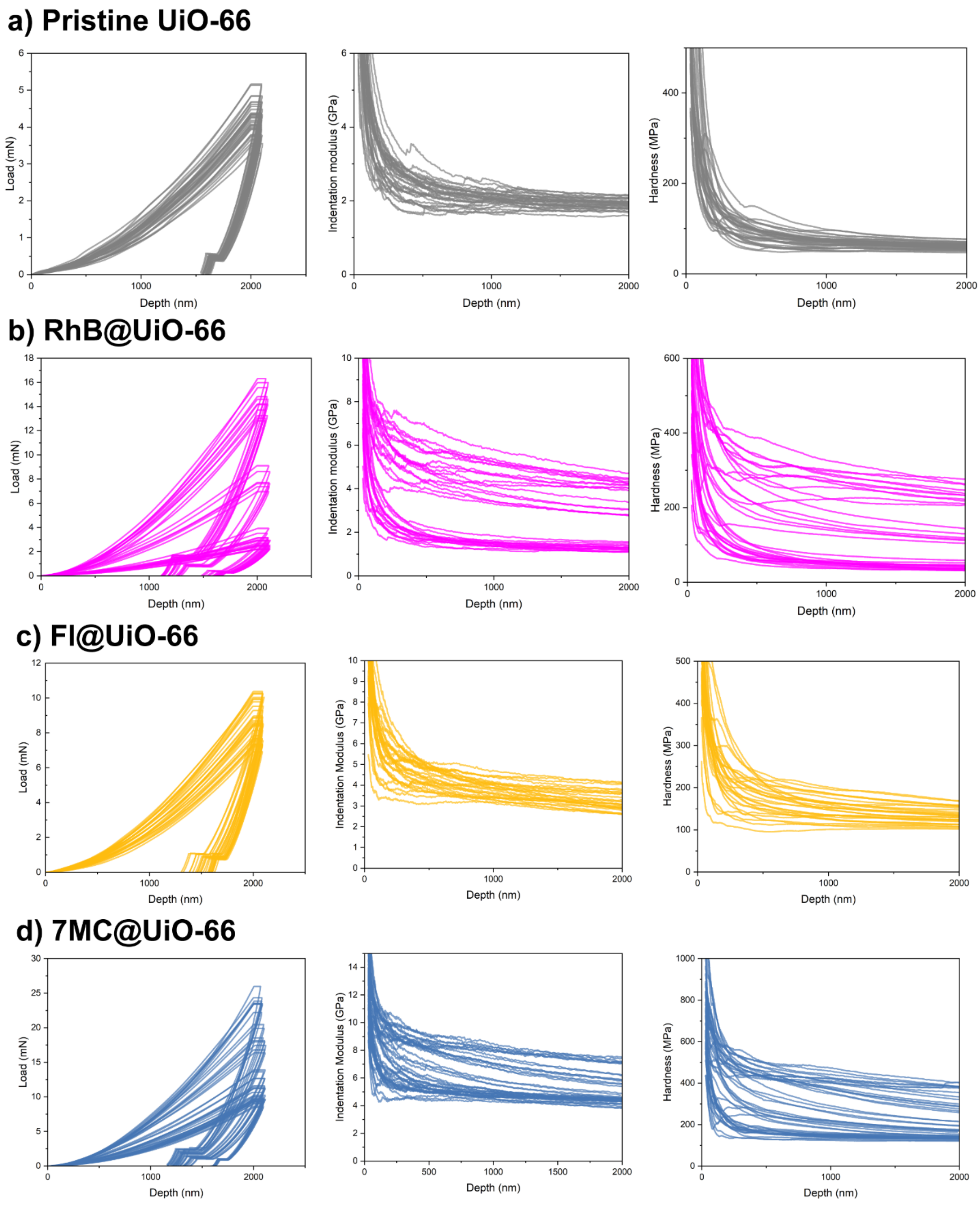

Figure S2: Nanoindentation tests employing the CSM method. Load-depth (left), indentation modulus-depth (centre) and hardness-depth (right) curves obtained for a) pristine monoUiO-66, b) RhB@monoUiO-66, c) Fl@monoUiO-66, and d) 7MC@monoUiO-66. Note: Horizontal segment in the load-depth curves at 90% unload corresponds to the thermal drift test segment.

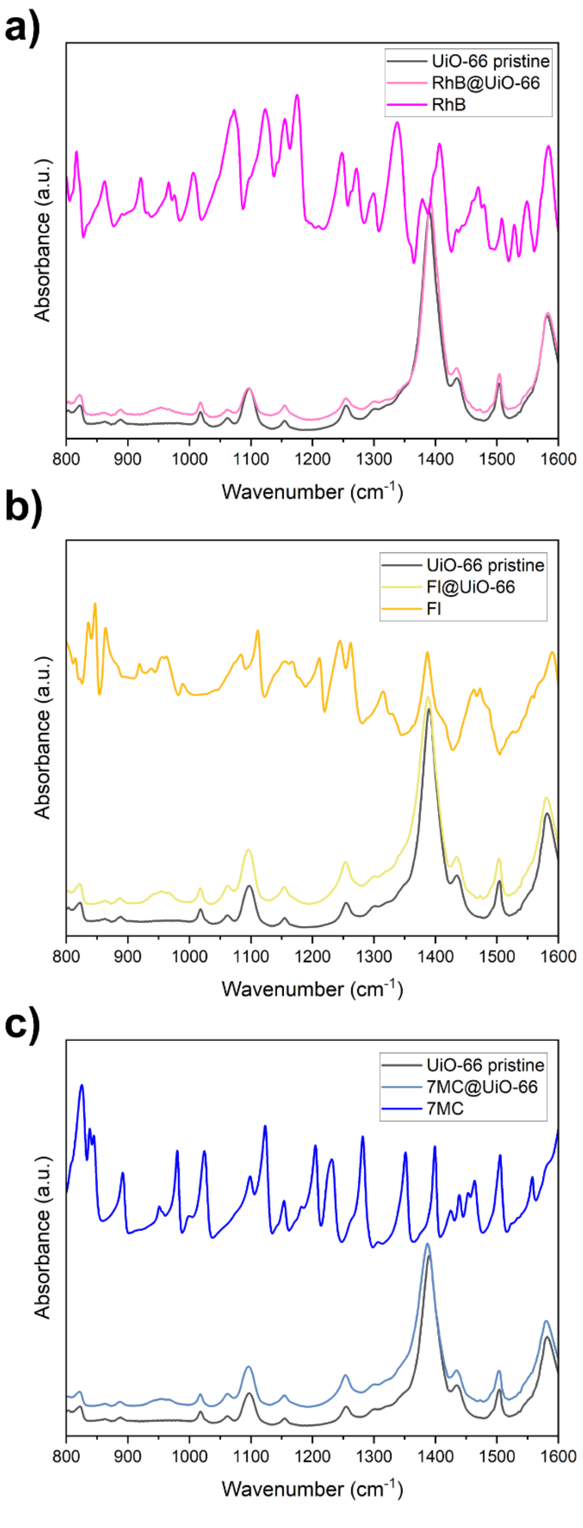

Figure S3: Comparison of ATR-FTIR spectra of a) RhB@monoUiO-66, b) Fl@monoUiO-66, and c) 7MC@monoUiO-66 against the pristine UiO-66 and the respective pristine dyes.

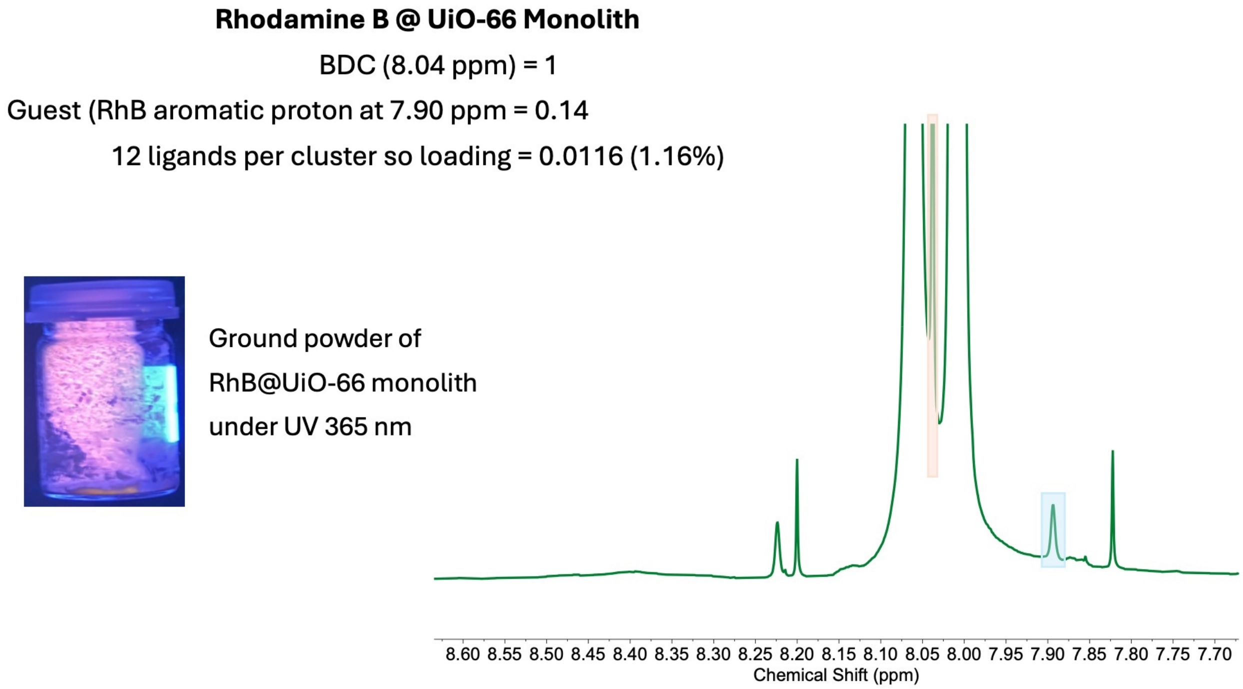

Figure S4: Solution ^{1}H NMR of RhB@UiO-66 where the host/guest peaks employed for integration are given in the inset. The guest loading calculated is 1 rhodamine B for every 86.2 pore. This analysis assumed that all guests are confined in MOF pores.

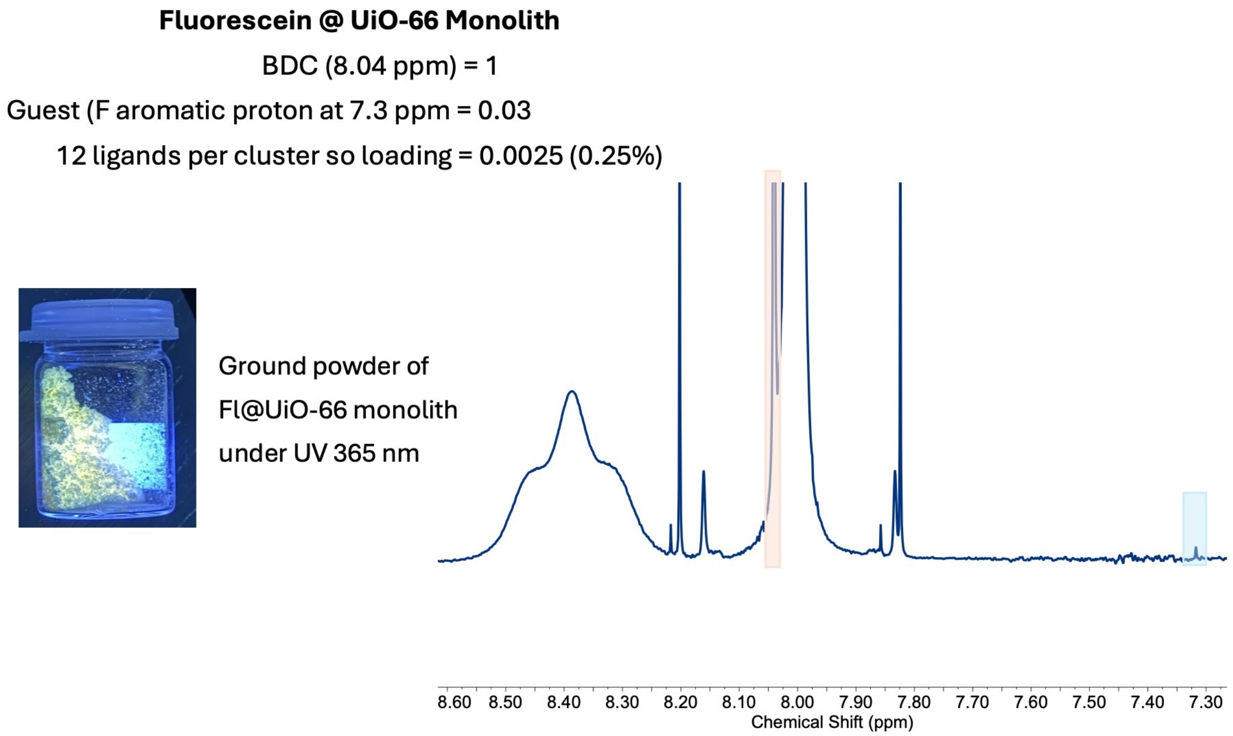

Figure S5: Solution ^{1}H NMR of Fl@UiO-66 where the host/guest peaks employed for integration are given in the inset. The guest loading calculated is 1 fluorescein for every 400 pore. This analysis assumed that all guests are confined in MOF pores.

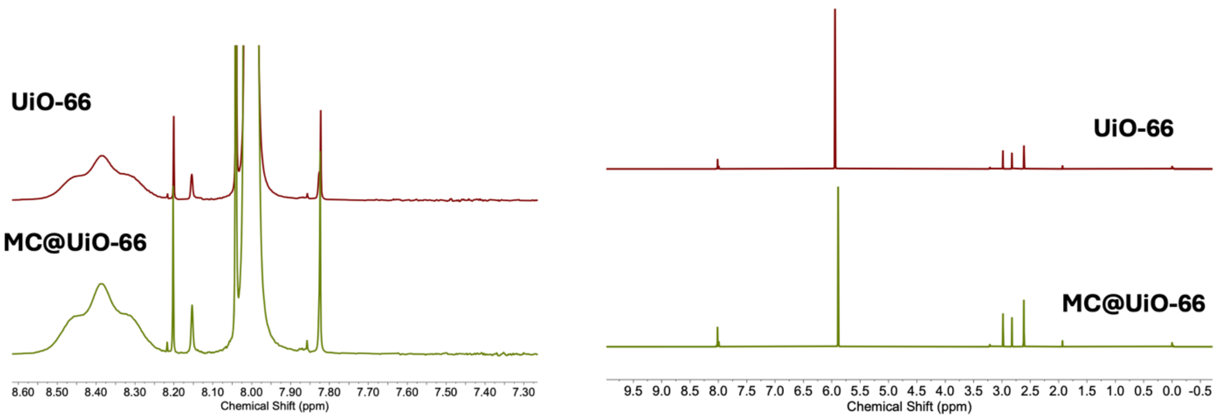

Figure S6: Solution ^{1}H NMR spectra of MC@UiO-66 are identical to the spectra of UiO-66, thereby the MC guest concentration was too low to be measured from the ground monoliths. 7-methoxycoumarin is expected to exhibit a doublet at ~6.9 ppm (absent here), corresponding to the proton in the ortho position relative to the methoxy group.

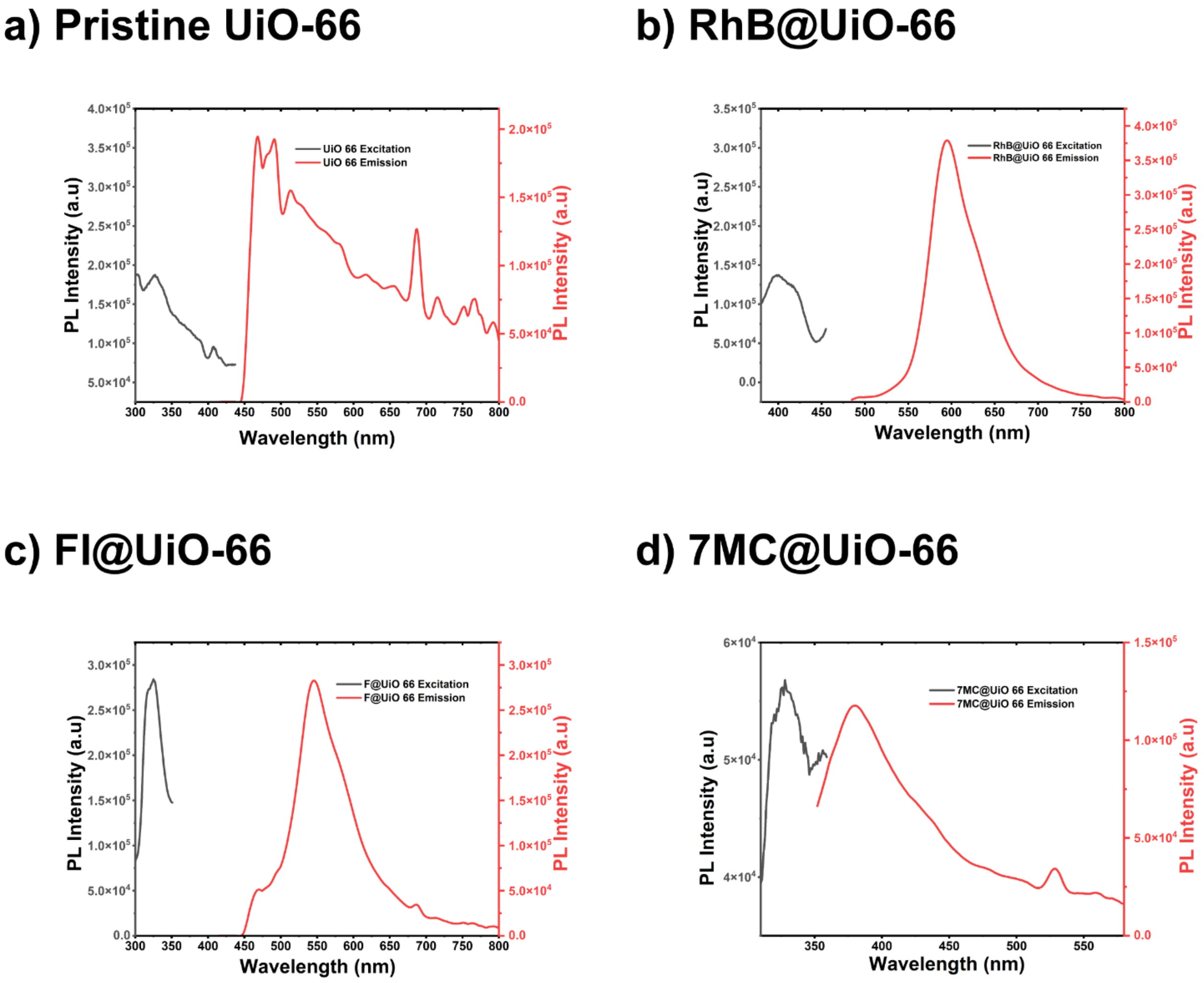

Figure S7: Photoluminescent excitation and emission spectra of the four monolithic systems of LG@monoMOF.

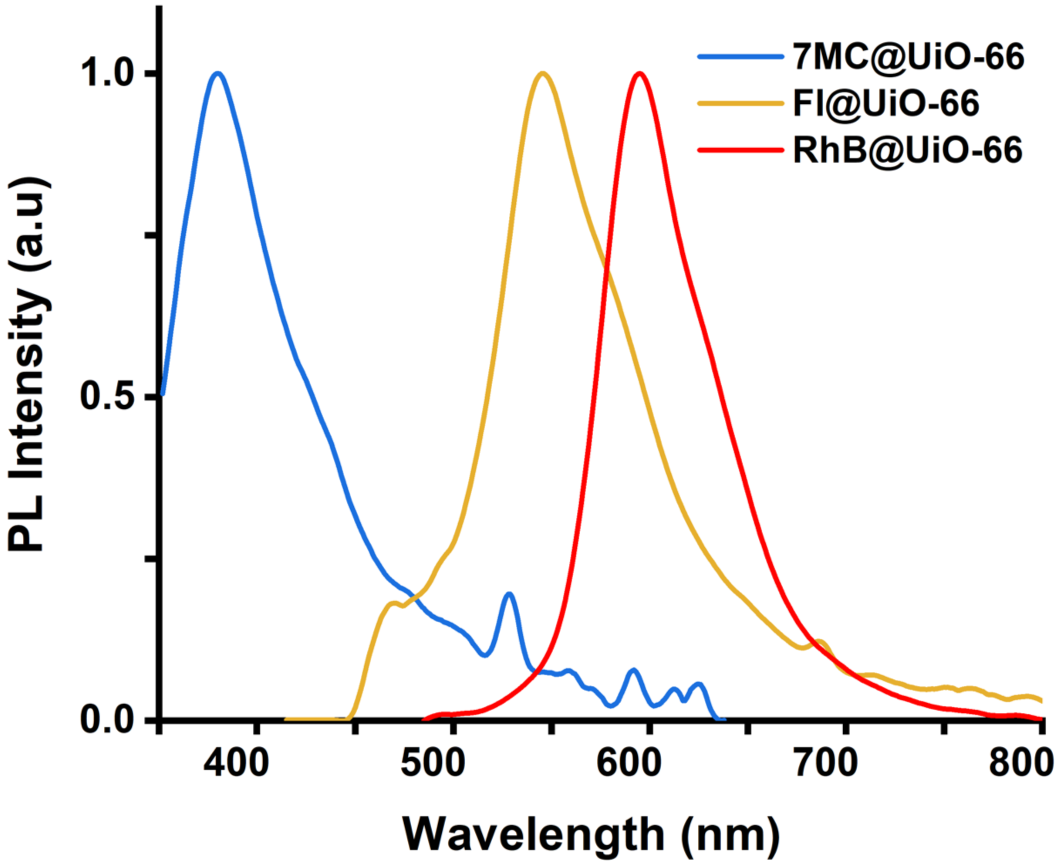

Figure S8: Comparison of the photoluminescent emission spectra of the three LG@monoMOF systems.

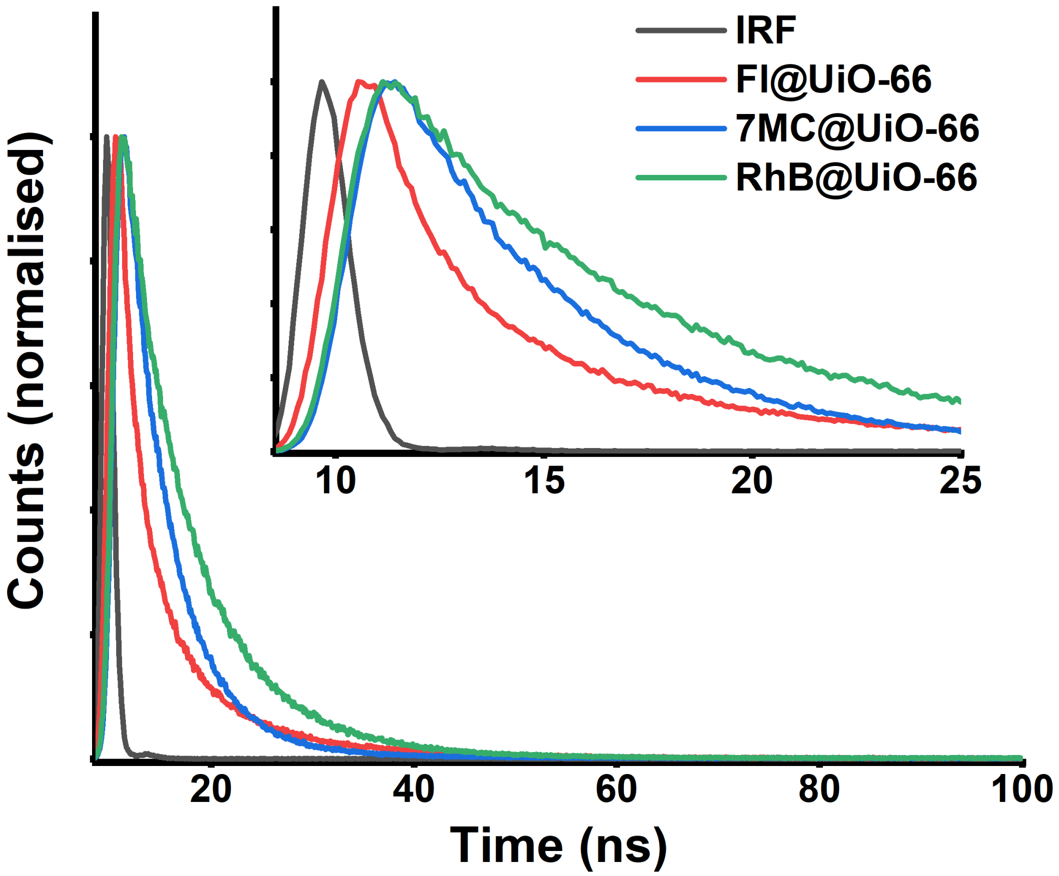

Figure S9: Lifetime emission spectra showing the decay curves of the three LG@monoMOF systems, determined from the TCSPC technique. The IRF curve corresponds to the instrument response function.

Table S1 Values of time constants (τ_i) derived from the time-resolved fluorescence decay lifetime measurements of the three LG@monoMOF systems. λ_{obs} is the observation wavelength.

Sample	λ_{obs} (nm)	τ_1 (ns)	τ_2 (ns)	τ_3 (ns)	χ^2
RhB@UiO-66	565	2.95	5.35	-	1.20
	595 (λ_{max})	5.34	9.19	-	1.16
	605	4.56	9.98	-	1.13
Fl@UiO-66	525	1.82	4.22	-	1.10
	544 (λ_{max})	2.51	4.57	-	1.00
	565	3.92	10.07	-	1.16
7MC@UiO-66	379	1.26	4.35	11.95	1.15
